# Primary Subcutaneous Hydatid Cyst with Palisading Granulomatous Reaction

**DOI:** 10.1155/2013/126541

**Published:** 2013-12-10

**Authors:** Noorah Almadani, Bader Almutairi, Ali H. Alassiri

**Affiliations:** ^1^Department of Pathology and Laboratory Medicine, King Abdulaziz Medical City, P.O. Box 22490, Riyadh 11426, Saudi Arabia; ^2^Department of Medical Imaging, King Abdulaziz Medical City, P.O. Box 22490, Riyadh 11426, Saudi Arabia

## Abstract

Palisading granulomatous reactions are prominent microscopic characteristics that are seen in many diseases. Isolated subcutaneous cystic echinococcosis is rarely documented. Palisading granuloma as a host immune reaction to *Echinococcus granulosus* in an isolated primary subcutaneous hydatid cyst has been reported only once before. In this report, we are describing a 53-year-old male who developed a slowly growing subcutaneous thigh mass. Light microscopy confirmed the presence of hydatid cyst. Further radiological workup for liver and lung has not shown any visceral hydatid focus.

## 1. Introduction

Cystic echinococcosis is an important tapeworm disease caused by *Echinococcus granulosus*, usually locating in liver or lungs, and subcutaneous location or extension is rare.

Palisading granulomatous reactions have been documented in several diseases. Furthermore, rare cases of echinococcosis that show palisading granulomatous reaction have been documented. Herein, we report an unusual clinical presentation with an uncommon microscopic feature of isolated subcutaneous cystic echinococcosis that shows prominent palisading granuloma.

## 2. Case Report

### 2.1. Clinical Features

**Figure 1:**
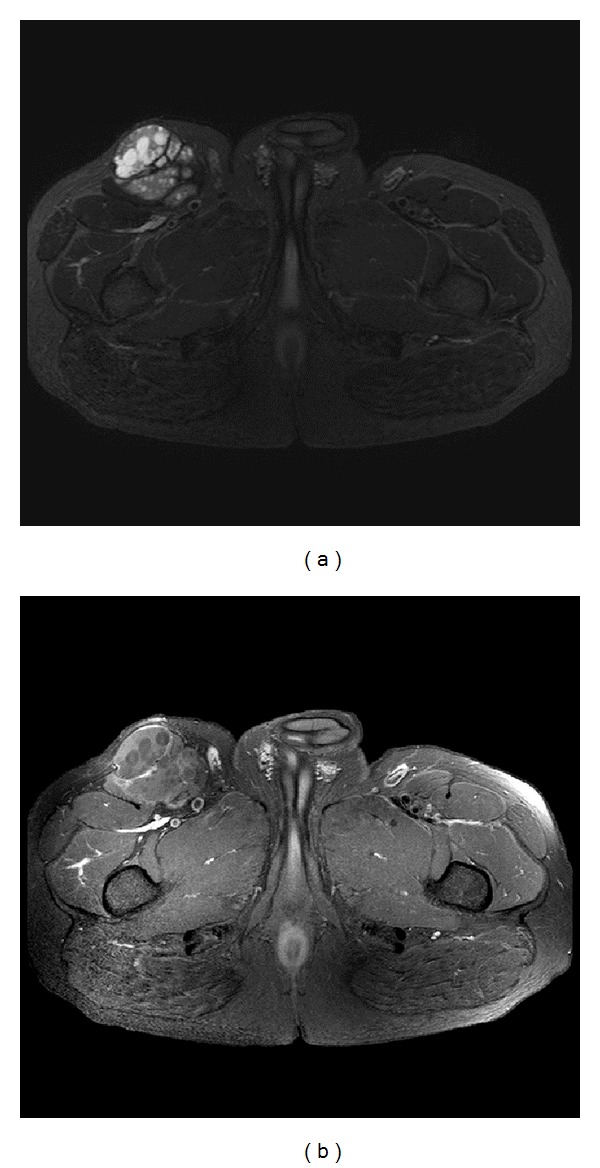
(a) Axial T2 weighed image with fat saturation, it demonstrates a complex cystic lesion arising from the right sartorius muscle. It contains multiple small rounded daughter cysts and internal fibrous septae. No signs of invasion to the adjacent structures to suggest an aggressive sarcoma. (b) Axial T1 weighted image with fat saturation and postcontrast enhanced study: it demonstrates lack of enhancement of the complex lesion. The small rounded daughter cysts are nonenhancing consistent with hydatid cyst. Overall, the lack of enhancement as well as absence of aggressive features favors hydatid disease over soft tissue sarcoma.

A 53-year-old male presented with a right thigh mass slowly growing over 10 years. Physical examination revealed a rounded, firm mass in the right upper thigh with no skin changes. Magnetic resonance imaging revealed a cystic well-circumscribed heterogenous soft tissue mass involving the mid-upper right thigh just inferior to the right inguinal ligament (Figures 1(a) and 1(b)). The mass measures 13.7 cm in maximum dimension. The patient underwent total excision of the mass with safe margins and his postoperative clinical course was uneventful.

### 2.2. Gross Features

Macroscopic examination of the received excisional biopsy showed a well-circumscribed subcutaneous cystic mass that measures 13.5 cm in maximum dimension covered by skeletal muscle tissue. The outer surface is intact and the section surface revealed multiloculated cystic mass containing thick yellow gelatinous material.

### 2.3. Microscopic Features

**Figure 2:**
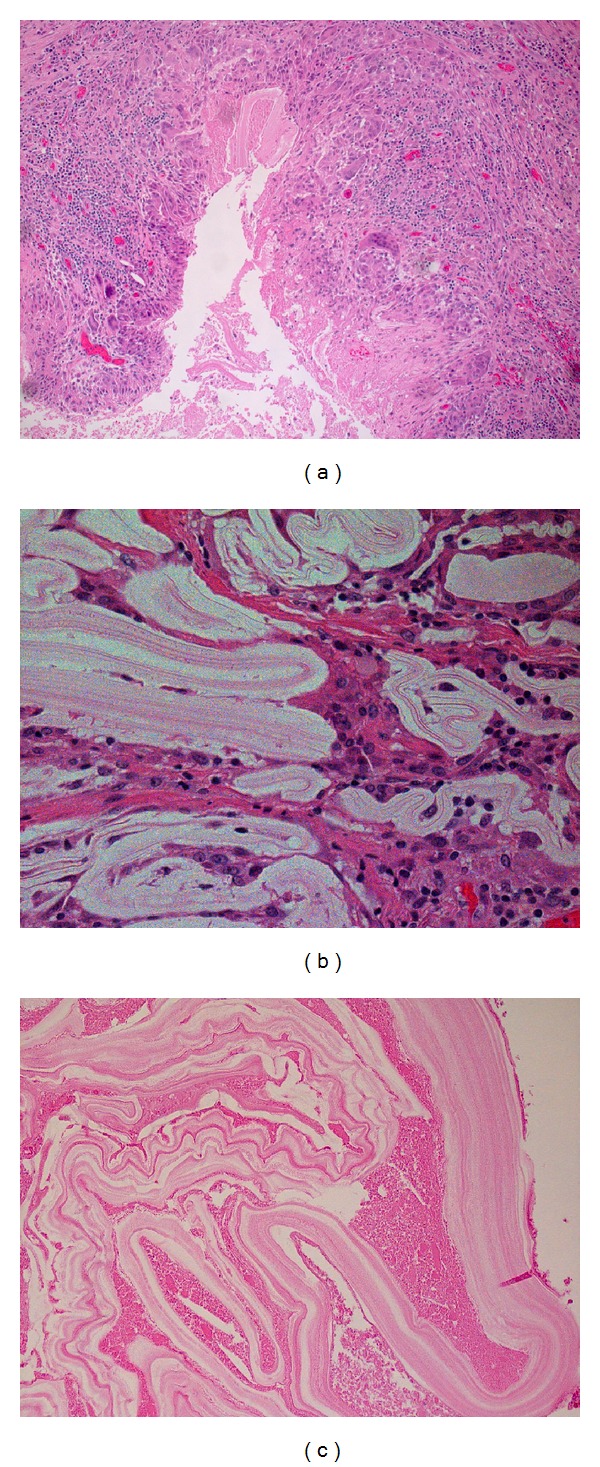
(a) A palisade of granulomatous reaction around membranous structures. (b) High power magnification displaying the laminated membranous structures eliciting an inflammatory response within the cyst wall. (c) High power magnification displaying the laminated characteristic of *Echinococcus* cyst wall.

Formalin fixed and paraffin embedded sections displayed variably sized cysts palisaded by granulomatous reaction (Figure 2(a)). The cyst contained PAS+ laminated membranous structures typical of *Echinococcus* cyst walls better appreciated on high magnification (Figures 2(b) and 2(c)). These are intensely stained with PAS stain.

### 2.4. Clinical Correlation

Subsequent serological assay for hydatid disease was positive. Computed tomography of chest, abdomen, and pelvis did not reveal any primary lesion.

## 3. Discussion

Hydatid disease is a parasitic infestation caused by the tapeworm *Echinococcus* granulosus. It is endemic in Saudi Arabia and the Middle East countries especially in rural areas in which humans live in close contact with sheep and dogs [1]. Established cystic *Echinococcus* has three main layers comprising the outer host layer or pericyst, the middle laminated membrane, which is the most important for diagnosis, and inner germinal layer. Scolices develop from outpouching of the germinal layer called brood capsules.

Although hydatid disease can develop anywhere in the human body, the liver is the most frequently involved organ (52–77%), followed by the lungs (10%–40%). The hydatid disease, as in our case, can remain asymptomatic for years or may develop serious complications as rupture, infection, anaphylaxis, and death.

Subcutaneous cystic echinococcosis is rarely reported and due to the diagnostic difficulties and mimicry of various subcutaneous cystic lesions, cystic echinococcosis should be considered in the differential diagnosis of any cystic mass [2]. Diagnosis of echinococcosis should be considered when slowly growing soft tissue mass is present in patients from rural area especially endemic countries. Before surgical excision or biopsy and extirpation of cyst, diagnosis of echinococcosis should be excluded to avoid leakage of cyst contents and the accompanying risks of anaphylaxis. Ultrasound is useful in diagnosis, showing the size, localization, and type of the cyst. The sensitivity of ultrasonography is 95% and if vesicular fibrils are present, the sensitivity of USS increases up to 100%. CT scan should be performed in suspicious cases or in order to determine the technique of surgery with demonstration of the relationship to adjacent organs.

Treatment is total surgical excision without opening the cyst. If the cyst cannot be excised without opening, the fluid contents should be aspirated, the laminated membrane should be totally excised, and the cyst pouch should be irrigated. The reported case had undergone wide excision. Identifying postoperative recurrence of the cyst in endemic regions is very difficult because the probability of formation of a new cyst is high [3].

Palisading granuloma is seen in various diseases such as granuloma annulare, necrobiosis lipoidica, and rheumatoid nodules. In the English literature, there is only one documentation of seven cases of subcutaneous cystic *Echinococcus* coexisting with palisading granuloma reaction all of which had a primary lesion elsewhere [4].

There are several reports of primary subcutaneous/soft tissue hydatid cyst [5–10], but our case report is the first documentation of a primary hydatid cyst associated with palisaded granulomatous reaction. This should alert the pathologist to at least consider hydatid disease in the differential diagnosis of a subcutaneous cystic mass with granulomatous reaction especially.
